# Atomic Force Microscopy Sidewall Imaging with a Quartz Tuning Fork Force Sensor

**DOI:** 10.3390/s18010100

**Published:** 2018-01-01

**Authors:** Danish Hussain, Yongbing Wen, Hao Zhang, Jianmin Song, Hui Xie

**Affiliations:** 1 State Key Laboratory of Robotics and Systems, Harbin Institute of Technology, 2 Yikuang, Nangang District, Harbin 150080, China; danishasefi@hit.edu.cn (D.H.); wenbo6210@hit.edu.cn (Y.W.); Haoz@hit.edu.cn (H.Z.); hitsjm@hit.edu.cn (J.S.); 2 Department of Mechatronics Engineering, National University of Science and Technology, H-12 Islamabad, Pakistan

**Keywords:** atomic force microscopy, sidewall, quartz tuning fork, cantilever

## Abstract

Sidewall roughness measurement is becoming increasingly important in the micro-electromechanical systems and nanoelectronics devices. Atomic force microscopy (AFM) is an emerging technique for sidewall scanning and roughness measurement due to its high resolution, three-dimensional imaging capability and high accuracy. We report an AFM sidewall imaging method with a quartz tuning fork (QTF) force sensor. A self sensing and actuating force sensor is fabricated by microassembling a commercial AFM cantilever (tip apex radius ≤10 nm) to a QTF. The attached lightweight cantilever allows high-sensitivity force detection (7.4% Q factor reduction) and sidewall imaging with high lateral resolution. Owing to its unique configuration, the tip of the sensor can detect sidewall surface orthogonally during imaging, which reduces lateral friction. In experiments, sidewalls of a micro-electro-mechanical system (MEMS) structure fabricated by deep reactive ion etching process and a standard step grating are scanned and the sidewall roughness, line edge roughness and sidewall angles are measured.

## 1. Introduction

Since its invention, the atomic force microscope has evolved into a versatile instrument with a wide spectrum of applications in the nano science and technology such as imaging of soft biological samples, atomic and subatomic level imaging, nano-manipulation and nano-metrology to name a few. In the standard AFM, a sharp tip at the apex of a microcantilever scans over the sample surface with an orthogonal *z*-servo to obtain the surface topography and various properties of the sample, such as elastic modulus and adhesion. However, this technique is limited in imaging sidewalls of the micro and nano structures for sidewall roughness (SWR) and critical dimension (CD) metrology. Sidewall imaging is important in the semiconductor industry during and after lithography to improve lithography process and device quality [1,2]. In addition, the SWR metrology is highly important because of its direct impact on the performance of the micro and nanoelectronics devices. For example, poor SWR can cause scattering losses in the microphotonic waveguides and performance degradation of fin field effect transistors (FinFETs) [3,4,5,6,7,8]. Similarly, during lithography, the SWR is measured to determine the effect of mask roughness, acid diffusion, shot noise and acid volatility on the quality of fabricated sidewall structures [9].

To enable AFM to access the sidewalls, shaped probes were used in the pioneering works [10,11]. In this approach, probe is vibrated in vertical and horizontal directions to effectively detect the surface slope in order to image the horizontal surfaces (top or bottom) and sidewall. However, image resolution on the sidewall is low due to big edge radius of the tip [12,13]. In addition, access to the high step sidewalls is limited due to less effective length of the tip (usually <1 μm). Similarly, methods with the tilted *Z*-scanner, probe or sample are also used to access sidewalls [14,15,16]. With these methods AFM capability to access high step sidewall has improved greatly. However, image resolution is dependent on the tilt angle and tip slip on the sidewalls can lead to the poor image quality and tip wear [17,18]. Dai et al. developed assembled cantilever probes by microassembling multiple AFM probes [19], which showed an excellent capability to access the sidewalls of the microstructures. In addition to the coventional silicon probe, the quartz tuning fork (QTF) force sensor is being widely used for AFM imaging due to high Q-factor, force sensitivity and high temperature stability, and that has enabled atomic as well as subatomic level imaging [20,21,22,23]. Sidewall imaging methods with the QTF force sensors are desired for flexible and high resolution sidewall scanning of micro/nano structures.

In this work, we report a self-sensing and actuating QTF force sensor based AFM method for non-destructive sidewall imaging with frequency modulation mode. The force sensor is fabricated by attaching an AFM cantilever on a prong of the QTF with the microassembly technique. Due to the lightweight quality of the attached AFM cantilever, the high-Q factor QFT force sensor allows high-sensitivity force detection. The lateral resolution of the QFT sensor is also improved in sidewall imaging because of its small tip apex (≤10 nm) [21]. Additionally, the structural design of the probe allows for orthogonal scanning of the sidewalls, which reduces lateral friction. Measurement principle and applications of the developed method for the sidewall roughness (SWR) and line edge roughness (LER) measurement are presented.

## 2. Preparation of the QTF Force Sensor

### 2.1. Determination of the Suspended Cantilever Length of the QTF

**Figure 1:**
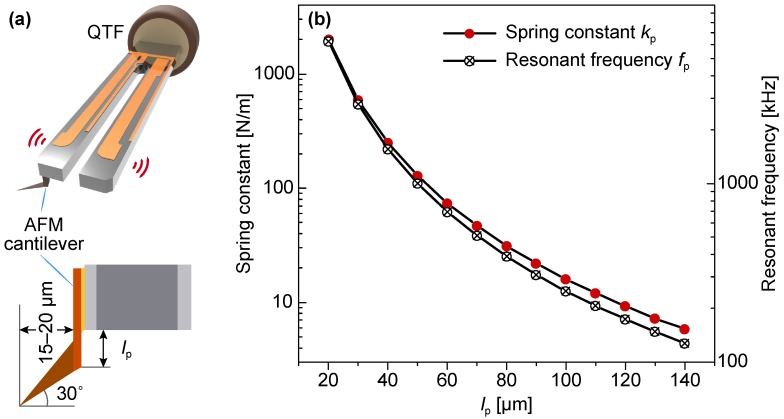
(**a**) configuration of the QTF force sensor; (**b**) variations in the spring constant and resonance frequency of the suspended cantilever as functions of the length ($l_{p}$) of the suspended cantilever ($l_{p}$).

The main issue with QTF (model: DS-26; fundamental frequency $f_{0}$: 32.756 kHz) sensor preparation is to glue a lightweight tip to achieve a high Q factor for high-sensitivity force detection. In this work, the cantilever of a contact mode AFM probe (ATEC-CONT, Nanosensors, Neuchâtel, Switzerland) is used as the QTF tip. It is specially manufactured with a protruding tip (tilt angle ∼30^∘^) that allows access to the bottom of the sidewall and provides a small tip apex radius (≤10 nm) for high-resolution image scan. In addition, the cantilever length (450 μm) is lager than the QTF thickness (∼300 μm), which facilitates probe microassembly on a tine of the QTF. Figure 1a shows the configuration of the QTF sensor. The backside of the cantilever is affixed to the outer side of the prong with the protruding tip parallel to the prong’s motion direction, which facilitate orthogonal probing on the sidewall (orthogonal scanning) when the QTF vibrates in its fundamental resonant mode. To access the sidewall of a deep trench, the cantilever (Figure 1a inset) is suspended from the prong of the QTF and its protrusion length ($l_{p}$) should be carefully selected in accordance with the following two conditions:

(1) The suspended cantilever should be stiff enough to be able to accurately detect tip-sample interaction with negligible bending deformations.
(2) Its fundamental resonance frequency should be much higher than that of the QTF to avoid coupling vibration noise.

The spring constant ($k_{p}$) and fundamental resonance frequency ($f_{p}$) of the suspended cantilever are determined by [24]:

(1) $$k_{p}=\frac{Ew_{p}t_{p}^{3}}{4l_{p}^{3}},$$

(2) $$f_{p}=\frac{1}{2\pi }\frac{5t_{p}}{2\sqrt{6}l_{p}^{2}}\sqrt{\frac{E}{\rho }},$$

where *E* and ρ are Young’s modulus and density of the probe’s material (Si), $w_{p}$, $t_{p}$ and $l_{p}$ are width, thickness and length of the suspended cantilever, respectively.

Figure 1b shows variations in the spring constant and fundamental resonance frequency of the suspended cantilever ($w_{p}=50$ μm and $t_{p}=2$ μm) with different lengths ($l_{p}$) from 20–140 μm. Simulation results demonstrate that, when $l_{p}<100$ μm, the fundamental resonance frequency of the suspended cantilever ($f_{p}>266.7$ kHz) is much higher than that of the QTF. Similarly, its stiffness increases rapidly from 16 N/m with the decreasing $l_{p}$. Considering the simulation results, $l_{p}$ was optimized in the range of 20–100 μm during the sensor assembly.

### 2.2. Microassembly of the QTF

**Figure 2:**
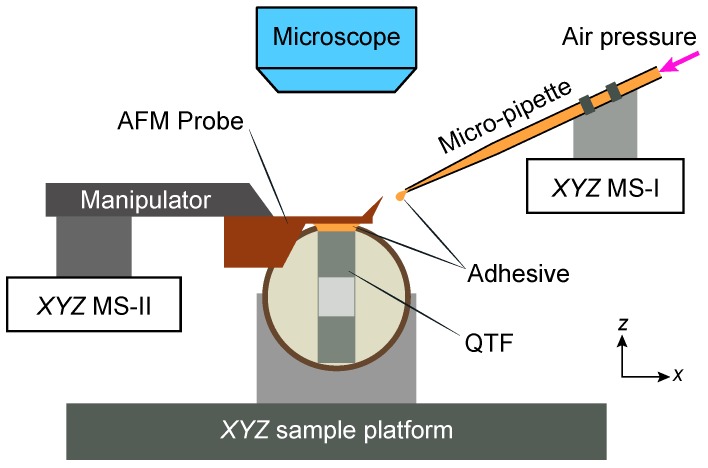
Schematic of the QTF force sensor prepartion with a dual-manipulator microassembly system.

The length, thickness and width of the used QTF prongs were measured as 3.729 mm, 0.515 mm and 0.302 mm under an optical microscope (20×), respectively. As shown in Figure 2, the AFM cantilever is affixed on one of the QTF prongs using a precision micromanipulation system with two micromanipulators. During the microassembly, the QTF is fixed on a custom fabricated sample stage supported by the *XYZ* sample platform, the AFM probe (with the probe chip) is fixed on the left manipulator (supported by a micropositioning stage (MS) *XYZ* MS-II) and a micropipette (aperture diameter 5 μm) is fixed on the right manipulator (supported by a micropositioning stage *XYZ* MS-I). Sufficient adhesive (two-component epoxy adhesive) was drawn into the micropipette before the manipulation by applying a suction pressure of -10 kPa for 30 seconds. The microassembly is manually performed under an optical microscope (20 ×) with the following procedure:

(1) The prong of the QTF is located under the optical microscope by moving the *XYZ* sample platform. At this point, the end of the prong is within the field-of-view of the microscope.
(2) By moving the micropositioning stage *XYZ* MS-I, the micropipette (filled with sufficient adhesive) is translated to slightly touch the desired bonding position at the end of the prong. Then, a small amount of adhesive is carefully deposited on the outer side of the prong by applying insufflation pneumatic pressure (5 kPa). In order to obtain an uniform distribution of adhesive at the bonding position, the micropipette is translated slightly from left to right during deposition.
(3) By moving the micropositioning stage *XYZ* MS-II, the AFM probe is positioned just above the bonding position with a desired length of the suspended cantilever. Then, the AFM probe is brought into contact with the deposited adhesive, and slight pressure is applied to compress the probe on the adhesive by moving the *z*-axis (MS-II).
(4) After the glue is fully cured (6–8 h), the cantilever is isolated from the chip of AFM probe by gently moving the left manipulator along the *z*-axis.

**Figure 3:**
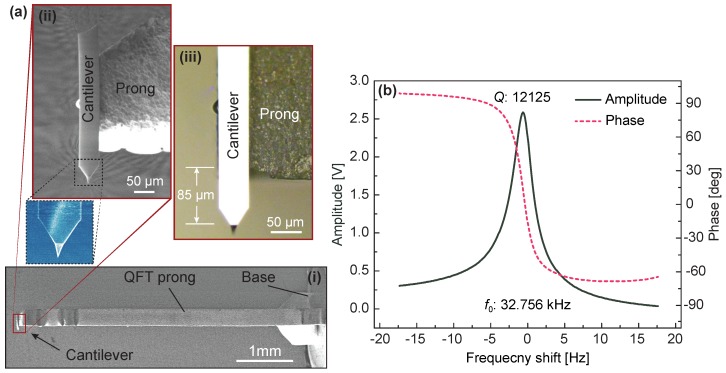
(**a**) QTF sensor; (**i**) SEM image, (**ii**) close-up SEM view, and (**iii**) optical microscope image; (**b**) frequency spectrum of a typical QTF force sensor.

Figure 3a shows scanning electron microscope (SEM) ((i) and (ii)) and optical microscopy ((iii)) images of the assembled QTF force sensor, where the length of the suspended cantilever is 85 μm. The Q-factor and resonance frequency of the sensors are measured with Nanonis electronics (SPECS Zurich GmbH, Zurich, Switzerland). As shown in Figure 3b, due to the lightweight of the attached cantilever, the sensor’s Q-factor is as high as 12,125, a reduction of only 7.4% as compared to the bare QTF (Q-factor: 13,097), which is a significant improvement over the previous results and allows high-sensitivity force detection without any techniques of mass balancing or Q-control [25,26].

## 3. Sidewall Imaging AFM System

### 3.1. Sidewall Imaging System

**Figure 4:**
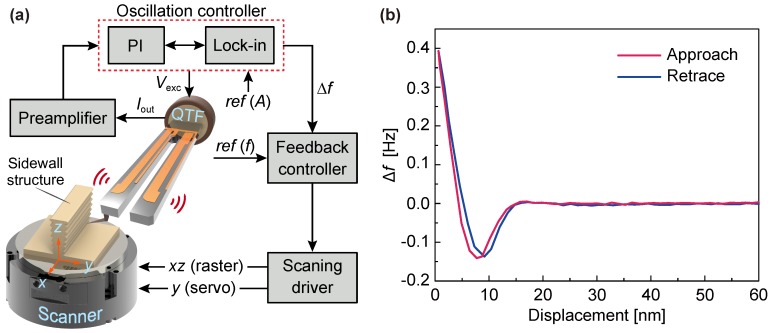
(**a**) schematic of the sidewall scanning method with the QTF force sensor. An *XYZ* scanner is used for image scan by moving sample. The QTF sensor is excited with a sinusoidal signal from an oscillation controller and current from the sensing electrode is collected by a transimpedance preamplifier. Two feedback loops are used to drive the sensor at a constant amplitude and regulate tip-sample distance; (**b**) frequency shift (Δf) of the QTF sensor as a function of tip-sample displacement measured on a silicon sidewall.

Figure 4a shows schematic of the method that is developed on a home-built AFM [27]. The QTF sensor is mounted on a *XYZ* motorized stage I (not shown here) for coarse positioning of the sensor. The sample (sidewall structure) is placed on a closed-loop *XYZ* scanner (NPXY60Z20, travel range: 60×60×20 μm) for image scan. The scanner is mounted on an *XY* motorized stage II (not shown here) for locating the scanning area of interest on the sample. An optical microscope (20×) and a charge coupled device (CCD) camera are used for locating the sample and aligning the force sensor. A Nanonis Oscillation Controller (Dual-OC4 & RC4, SPECS Zurich GmbH, Zurich, Switzerland) is used for sensor excitation ($V_{\mathrm{exc}}$), which is referenced to the pre-amplified (KolibiPreamp, SPECS GmbH, Berlin, Germany) sensor current output ($I_{\mathrm{out}}$). The prongs of the electrically excited QTF vibrate in phase and out of phase alternately (fundamental mode). The protruding tip (attached) parallel to the direction of vibration of the tines can probe the sidewalls orthogonally. When the tip is for away from the sidewall, the piezoelectric current in the sensing electrode (which is generated due to the deformation of the tines) is in phase with the excitation signal. The tip–sample interaction cause a frequency shift of the generated current ($I_{\mathrm{out}}$), which is measured by the phase locked loop (PLL, integrated in the Nanonis Oscillation Controller). In the setup, the frequency modulation (FM)-AFM is implemented with two feedback loops: (i) constant amplitude control loop within the Oscillation Controller using a proportional-integral (PI) controller, and (ii) frequency feedback control loop within the computer. The former is used to drive the QTF sensor at a constant oscillation amplitude (Ref (*A*)), and the latter controls the tip sampling distance (*y*-axis) by regulating the frequency offset Δf to the user set Ref (*f*).

Figure 4b shows the curves of the frequency shift (Δf) of the QTF sensor (self-oscillation amplitude: ∼15 nm), which is a function of tip-sample displacement measured on a silicon sidewall. During the approach, attractive forces lead to a negative frequency shift. As the tip gets closer to the surface, repulsive forces dominant the tip-sample interaction and drive the frequency shift in the positive direction. To get stable control of image scan, the frequency shift setpoint is set as 0.05 Hz (in the repulsive zone), which is far from the inversion of attractive and repulsive behaviours.

### 3.2. Sidewall Imaging Protocol

**Figure 5:**
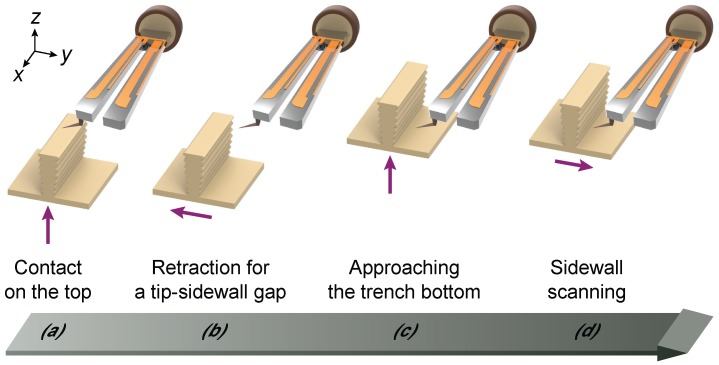
Protocol for the sidewall scanning. (**a**) servo on the *z*-axis is started to bring the tip into contact with the top surface (near the trench); (**b**) sample motion to locate the tip over the trench; (**c**) servo on the *z*-axis to enter the trench; and (**d**) servo on the *y*-axis for the image scan.

Deep sidewalls can be imaged with a high resolution due to the long $l_{p}$ and sharp tip of the QTF sensor. A systematic protocol is proposed to access the deep trench sidewalls and its main steps are as follows:

(1) When the tip is near the surface, *z*-servo is started with small vibration amplitude. The tip contacts the top surface and servo becomes stable as shown in Figure 5a.
(2) The *z*-servo is stopped and the sample is moved hundreds of nanometers downwards by the scanner to prevent damage to the sample or tip. Then, the sample is moved on the *y*-axis of the scanner to form an appropriate tip-sidewall gap, so that the tip is over the deep trench, as shown in Figure 5b.
(3) *z*-servo started to raise the sample upwards and the cantilever with the tip penetrates the trench (Figure 5c). In this case, the tip can touch the trench bottom if the trench is shallower than the *z*-travel distance of the scanner (while considering the initial *z*-position) or it can not touch the bottom if the trench is deeper than the *z*-travel distance.
(4) Finally, servo is switched from the *z*-axis to *y*-axis and the tip is approached on the sidewall (Figure 5d). The target sidewall is then scanned with the fast and slow scans on *x*- and *z*-axes, respectively.

Once the scan is completed, the tip is retracted from the sidewall and moved few micrometers above the top surface. The above protocol is repeated to scan other sidewall surfaces.

### 3.3. Algorithms of Sidewall Roughness and Line Edge Roughness

Sidewall roughness (SWR) is defined as the variations in the texture of the sidewall surface with respect to a reference plane (mean-line). In the statistical analysis of the SWR, the accuracy depends on the sampling length (*l*), sampling interval (Δ) and instrumental resolution [28]. Therefore, for higher accuracy, Δ and *l* are taken as equal to the scanning step length and total length of each extracted AFM profile, respectively. It can be measured along a single line scan or along several parallel lines. SWR (in the semiconductor industry) is usually evaluated as the standard deviation of the distance between a reference line [29]:

(3) $$\begin{array}{c} R_{q}=\sqrt{\frac{1}{n}\sum_{i=1}^{n}{\left(y_{i}-\bar{y}\right)}^{2}}, \end{array}$$

where $\bar{y}=\frac{1}{n}\sum_{i=1}^{n}|y_{i}|$ and *n* is the total number of data points in the evaluation and $y_{i}$ are the data points representing the depth of each point in the servo direction (*y*-axis).

Similarly, the Line Edge Roughness (LER, $R_{e}$) is defined as [30]:

(4) $$R_{e}=3\sqrt{\frac{1}{n}\sum_{i=1}^{n}{\left(y_{i}-\bar{y}\right)}^{2}}.$$

## 4. Experiments and Discussion

### 4.1. Sidewall Scanning of a MEMS Structure

**Figure 6:**
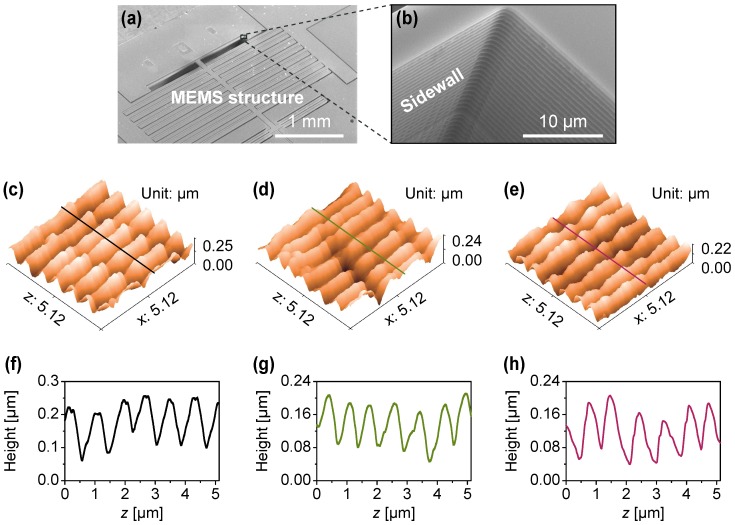
Sidewall imaging of a DIRE fabricated MEMS structure with the QTF force sensor. (**a**) SEM image of the MEMS structure and (**b**) the enlarged sidewall; (**c**–**e**) sidewalls’ topographies at different regions; and (**f**–**h**) height profiles through the lines indicated in (**c**–**e**), respectively.

Sidewalls of a micro-electro-mechanical system (MEMS) nanopositioning platform fabricated by deep reactive ion etching process (DRIE) was firstly scanned to demonstrate the performance of the developed method. As shown in Figure 6a, the MEMS structure has comb-drive actuators to drive the stage on a plane [31]. Each comb-drive actuator is featured with deep trenches and microcomb structures. In order to evaluate the sidewall roughness, different scan regions were selected on the deep trench sidewall (Figure 6b). The sample is placed with the sidewalls parallel to the fast scan direction on the *x*-axis. Sidewall scan is performed using the protocol described in Section 3.2. After one of the selected regions (sidewalls) is completed, the tip is retracted and aligned to the next. This process is repeated for sidewall scan of different regions.

Figure 6c–e show examples of the AFM topographies of the sidewalls scan of the MEMS structure with 256 lines of 256 points (scan area: 5.12 μm × 5.12 μm, step length: 20 nm). Figure 6f–h show height profiles of the sidewalls scanned through the lines indicated in Figure 6c–e, respectively. All images and respective profiles show that the sidewalls of the MEMS structure have waviness along the length of the features (*x*-axis) that are generated during the deep reactive ion etching (DIRE) process.

Ten height profiles along the *z*-axis on each AFM image were extracted at equal intervals (along the *x*-axis) for statistical analysis and SWR is calculated using Eqaution (3). Table 1 shows the statistical results of the sidewalls selected from different regions of the MEMS structure. Mean $R_{q}$ and $R_{\max}$ (the height difference between the largest peak and the largest valley in each profile) values are calculated as 41.36 nm and 282.48 nm, respectively. However, the data shows the variation in the roughness is different from region to region on the structure.

### 4.2. Sidewall Scanning of a Step Grating

**Figure 7:**
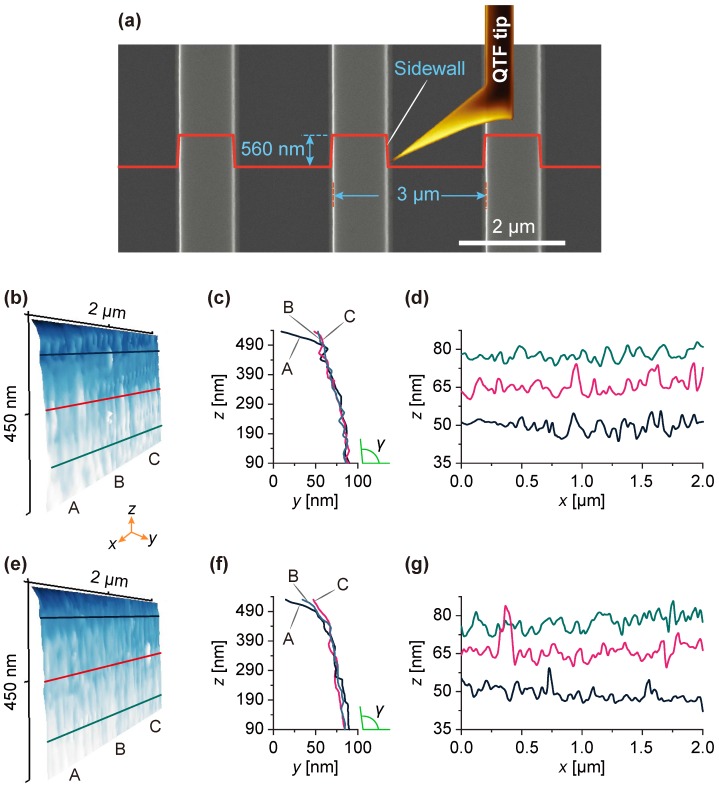
Sidewall imaging of a step grating with the QTF force sensor. (**a**) SEM image of the grating and schematic of scanning; (**b**) topography of one scan area on the sidewall; (**c**,**d**) profiles along the *z*-axis through points A, B, and C and lines indicated in (**b**), respectively; (**e**) topography of the second scan area on the sidewall; (**f**,**g**) profiles along the *z*-axis through points A, B, and C and lines indicated in (**e**), respectively.

To demonstrate the capability of sidewall imaging of nanostructure, sidewalls of a step grating TGZ3 (NT-MDT, Moscow, Russia) were scanned. The grating has a top width, step height and pitch of about 1 μm, 560.0±2.6 nm and 3.00±0.01 μm, respectively (provided by the manufacturer). The grating was aligned with the sidewalls parallel to the *x*-axis (fast scan direction). As illustrated in Figure 7a, the QTF sensor can reach the bottom of the narrow trench (∼2 μm width) and scan the footing of the sidewall due to the tilted protruding tip. Once the grating top surface was detected, the sidewall scan begun near the top edge. Ninety lines of 200 points were recorded on each scan area (2.00×0.45 μm) of the sidewalls. Figure 7b,e show the reconstructed AFM topography images of the TGZ3 sidewall. Figure 7c,f illustrate line profiles along the slow scan direction (*z*-axis) through the points A, B, and C indicated in Figure 7b,e, respectively. Close to the top part, profile A is obviously different from other two profiles, which indicates that the grating is tilted because of the sum of the mounting error and out-of-plane motion of the scanner on the *x*-axis. These line profiles are used to calculate the sidewall angle γ. Averaged from 200 measurements on slow scan lines, the sidewall angles summarized in Table 2 (the segments are defined along the *x*-axis) with consideration of the mounting angle error of the grating. Figure 7d,g are line profiles along the lines indicated in Figure 7b,e, respectively. The height profiles along the *x*-axis were extracted at equal intervals (along the *z*-axis) for statistical analysis of the LER. The LER measurement results on these lines are summarized in Table 3.

We have measured the sidewall roughness of a MEMS microcomb structure and LER as well as sidewall angles of the TGZ3 AFM standard grating. The reported method has the capability to measure sidewall features such as shape, SWR, sidewall angle and LER. The obtained data is valuable for the engineers and nano technologists who are working on the micro and nano-fabrication. Sidewall imaging with the QTF sensor can provide a flexible and cost-effective solution for CD metrology and can have several applications such as monitoring of etching parameters to improve the process [30] and sidewall roughness metrology of the optical waveguides [32].

## 5. Conclusions

We developed an atomic force microscopy sidewall imaging method with a quartz tuning fork force sensor, which is fabricated by attaching a commercial probe with a protruding tip (apex radius ≤10 nm) to the end of the prong. Effects from the tip slip on the sidewall imaging is avoided with this method due to the effective orthogonal scan manner, which allows high-resolution sidewall scanning and precise roughness measurement. Sidewalls of a MEMS structure and a step grating have been scanned, and sidewall roughness (line edge roughness) is characterized. Experimental results demonstrate the capability of the proposed method in accurately imaging and measuring the sidewall roughness of the micro and nanostructures. As the QTF sensor is self-sensing and actuating, so it can be integrated into any commercial AFMs with addition of suitable electronics and software algorithms. The presented method has potential for sidewall characterization during different steps of the lithography process and MEMS industry in optimizing fabrication variables.

References

## Figures and Tables

**Table 1:** Sidewall roughness (SWR) evaluation of the MEMS structure.

Region	$R_{q}$ (nm)	$R_{\max}$ (nm)
1	42.64	362.11
2	49.53	266.25
3	31.91	219.07
Mean	41.36	282.48

**Table 2:** Sidewall angle measurement results of the grating (Unit: Degree).

Segments	1/4–1/2	1/2–3/4	0–1/4	0–1/2	0–3/4
γ(±3σ)	85.52±0.77	84.18±0.83	86.62±0.84	85.64±0.82	85.03±0.72

**Table 3:** LER measurement results of extracted lines on the sidewall of the grating (Unit: nm).

Lines	Sidewall I			Sidewall II		
	Green	Pink	Black	Green	Pink	Black
LER (3σ)	7.57	10.29	8.91	7.75	10.98	7.57
